# Factors Associated With Off-Label Oncology Prescriptions: The Role of Cost and Financing in a Universal Healthcare System

**DOI:** 10.3389/fphar.2021.754390

**Published:** 2021-10-19

**Authors:** Noa Gordon, Daniel A. Goldstein, Boaz Tadmor, Salomon M. Stemmer, Dan Greenberg

**Affiliations:** ^1^ Department of Health Policy and Management, School of Public Health, Faculty of Health Sciences, Ben-Gurion University of the Negev, Be'er-Sheva, Israel; ^2^ Institute of Oncology, Davidoff Cancer Center, Beilinson Hospital, Rabin Medical Center, Petah Tikva, Israel; ^3^ Sackler Faculty of Medicine, Tel-Aviv University, Tel-Aviv, Israel; ^4^ Clalit Health Services, Tel-Aviv, Israel; ^5^ Beilinson Hospital, Rabin Medical Center, Petah Tikva, Israel

**Keywords:** cancer, off-label, cost, reimbursement, universal healthcare system

## Abstract

**Purpose:** Various solutions have been put forward for prescribing and reimbursing treatments outside their registered indications within universal healthcare systems. However, most off-label oncology prescriptions are not reimbursed by health funds. This study characterized the financing sources of off-label oncology use and the predictors of the decision to forego treatment.

**Materials and Methods:** All 708 off-label oncology requests submitted for approval in a large tertiary cancer center in Israel between 2016 and 2018 were examined for disease and patient sociodemographic characteristics, costs and financing sources, and the factors predicting actual off-label drug administration using multivariate logistic regression analysis.

**Results:** The mean monthly cost of a planned off-label treatment was ILS54,703 (SD = ILS61,487, median = ILS39,928) (approximately US$ 15,500). The main sources of funding were private health insurance (25%) and expanded access pharma company plans (30%). Approximately one third (31%) of the requests did not have a financing source at the time of approval. Of the 708 requests, 583 (or 82%) were filled and treatment was initiated. Predictors for forgoing treatment were the impossibility of out-of-pocket payments or the lack of a financing solution (OR = 0.407; *p* = 0.005 and OR = 0.400; *p* < 0.0005).

**Conclusion:** Although off-label recommendations are widespread and institutional approval is often granted, a large proportion of these prescriptions are not filled. In a universal healthcare system, the financing sources for off-label treatments are likely to influence access.

## Introduction

Regulatory registration and approval are mandatory for new drugs to be marketed and for public and private health insurance reimbursement. *Off-label drug use* is defined as the prescription of an approved drug for a purpose not indicated in the marketing authorization. This includes its use to treat other conditions, age groups, dosages, or routes of administration (Saiyed et al., 2017; Wittich et al., 2012).

Off-label drug use in oncology is widespread, with estimates of up to 75% (Conti et al., 2013; Joerger et al., 2014; Hamel et al., 2015; Kalis et al., 2015; Eaton et al., 2016). There are several reasons for its growing prevalence in recent years. The first is that the regulatory approval process is expensive and lengthy. If a drug is unauthorized but used *de facto*, pharmaceutical companies have no major incentive to expand its registration and marketing authorization. This is especially germane to off-patent drugs and to rare indications where Phase III randomized controlled trials may not be feasible or economically viable (ASCO, 2006). Second, even if a drug is in clinical development, aiming for registration for a new indication, the process until final authorization is granted is lengthy. In the interim, new evidence supporting off-label use might emerge and even be included in clinical practice guidelines. Third, with life-threatening and terminal illnesses such as cancer, patients and physicians look to unapproved treatments with limited supporting evidence after standard therapies have been exhausted. This “off-evidence” use may benefit patients, based on the reasoning that different cancer indications share the same genetic or molecular characterizations. Thus, in recent years, with the increasing incorporation of personalized or tailored medicine into clinical practice, off-label use prevalence has also grown. Finally, off-label drug use may provide real hope for effective treatments that might emerge in the future. This is defined as its “option value” (Garrison et al., 2017).

New treatments revolutionized cancer care in recent years, including targeted therapies and immunotherapy. Although many have high prospects, others will be proved to have limited value in the long term (Goldstein et al., 2016). At the same time, cancer drug prices at product launch are steadily increasing (Bach, 2009; Elkin and Bach, 2010), and continue to rise after market entry, regardless of competition and market volume (Bennette et al., 2016; Gordon et al., 2017). Hence, reimbursement of new oncology treatments with questionable value is a key issue, both in market-based and universal healthcare systems (Garrison et al., 2018). In the United States for example, the Centers for Medicare and Medicaid Services (CMS) limit coverage for off-label indications to those listed in specified compendia (ASCO, 2006; Abernethy et al., 2009; Green et al., 2016) while private insurers provide off-label reimbursement, depending on the indication and supporting evidence.

In universal healthcare systems, new treatments usually compete for a share in limited budgets. Including reimbursement for off-label drugs further complicates decision-making in that public reimbursement is usually more restrictive and depends on the national health insurance legislation. In Ontario (Canada), for instance, off-label treatments are only reimbursed for severe conditions where there are no alternative treatments (Rawson and Chhabra, 2018). In European countries, coverage policies vary (Weda et al., 2017). For example. in France reimbursement is approved when there is no authorized alternative (Natz and Campion, 2012; Weda et al., 2017). In Italy, off-label prescription is legal if there is adequate evidence for safety and efficacy, however public reimbursement is available only for certain drugs included in specific lists, updated as clinical evidence accumulate (Gozzo et al., 2020). In Germany and in Japan, expert commissions are established to approve specific off-label treatments use and reimbursement (Weda et al., 2017; Bun et al., 2020).

In Israel, drugs can only be legally prescribed for their registered indications. However, regulation 29 of the Pharmacist’s Regulations (1986) notes several exceptions concerning the use of unlicensed medical products and the unapproved indications of licensed medical products, including off-label use. The Institutional Drug Committees (IDCs), which are established within medical centers, provide approval if the use of the drug has been shown to be imperative and there are no other viable alternatives. As for reimbursement, the Israeli National List of Health Services (NLHS) specifies the drugs and other health technologies and services to which all residents are entitled. New treatments are recommended by a public national advisory committee and reviewed in a comprehensive process that includes clinical, economic, social and ethical factors (Shani et al., 2000). The NLHS stipulates a mandatory basic “basket” of health services that Health Maintenance Organizations (HMOs) in Israel are obligated to provide. Each HMO may add services to the basic basket. HMO exception committees discuss reimbursement of individual treatment requests that are not included in the NLHS. New health technologies are rapidly reviewed for inclusion each year, with a very good coverage of new drugs (Greenberg et al., 2009; Ribalov et al., 2016). However, since one prerequisite is approval by a major regulatory agency (e.g., the U.S. FDA) and registration in Israel, off-label use is generally not publicly reimbursed (Hammerman et al., 2011). Instead, off-label drug use is largely financed through commercial health insurances, charitable organizations, expanded access plans that are offered by pharmaceutical companies, or paid out-of-pocket by patients and their families.

Research has examined the frequency of off-label use, toxicity, and outcomes (Conti et al., 2013; Eaton et al., 2016; Herrero Fernandez et al., 2019). However, to the best of our knowledge, no study to date has assessed the economic burden and financing sources of these treatments. The current study was conducted in a large, tertiary cancer center in Israel. The objective was to describe the costs and range of financing strategies of off-label treatments in oncology and to identify the reasons why approved off-label treatments are not initiated.

## Methods

All consecutive off-label requests approved between January 2016 and December 2018 by the Institutional Drug Committee (IDC) at Rabin Medical Center (RMC; Petah Tikva, Israel) were examined. The RMC is a 1,100-bed academic tertiary hospital and one of the largest referral centers in the country, treating approximately 20% of all cancer patients in Israel. The RMC is owned by Clalit Health Services, the largest public health insurer in Israel that provides coverage to approximately 52% of the Israeli population. The IDC reviews each request based on the available evidence to weigh the risks and potential benefits. All records are retained by the hospital pharmacy. Only injectable, oncology off-label drug requests were included, since these are reviewed by the IDC and administered in the hospital’s outpatient clinic. We excluded requests for patients who died less than 60 days after request approval to minimize the bias of performance status and life expectancy on off-label treatment initiation.

For each off-label request, we collected the drug and indication information, disease characteristics, and intended financing source for the treatment. Supporting evidence was assessed for each off-label treatment according to the ESMO Magnitude of Clinical Benefit Scale (ESMO-MCBS) version 1.1 (Cherny et al., 2017) and the ESMO Scale for Clinical Actionability of molecular Targets (ESCAT) (Mateo et al., 2018). The data were then categorized into three groups: sufficient evidence (ESMO-MCBS grade A-B, 5-4), limited evidence (ESMO-MCBS grade C, 3-1; ESCAT tier II-IIIA) and inadequate evidence (no supporting clinical trials; ESCAT tier IIIB-IV). Patient sociodemographic characteristics and in-depth disease information were collected from electronic medical records. The distance from the medical center and the socioeconomic status (SES, ranging from 1 to 10 by deciles) were calculated according to the patient’s home address. We extracted off-label drug dispensing dates and dosages from the pharmacy dispensing database. We then calculated the monthly cost based on drug price lists published by the Ministry of Health (Ministry of Health, 2016). Costs are presented in Israeli Shekels (ILS), at an exchange rate of ILS3.50 to $1.00US.

The patient, disease, costs, and financing variables were subjected to descriptive statistics. A one-way ANOVA was used to compare the mean monthly costs between different groups. Univariate logistic regression analysis was performed to estimate the relationship between the independent variables and treatment initiation. Multivariate regression was used to identify factors predicting actual treatment initiation. Age, gender, and all variables that were found to be significantly distributed differentially across groups were entered into the multivariate logistic regression in one step. Statistical significance was set a-priori at *p* ≤ 0.05. The data were analyzed using IBM SPSS Statistics for Windows, version 26 (IBM Corp., Armonk, N.Y., United States). This study was approved by the RMC ethics committee (0068-16-RMC); participant’s consent was not required.

## Results

### Off-Label Requests and Patient’s Characteristics

The IDC approved 1,216 requests between January 2016 and December 2018. Of these requests, 814 were for injectable oncology off-label drugs 106 requests were for patients who died less than 60 days after approval and were excluded from the analysis, leaving 708 requests for 618 patients. The mean patient age was 62 years (range 19–95); 58% were female. The median SES decile was 7 (range 1–9). Most patients lived in the center of Israel with a mean distance of 30 km from the RMC (range 3–344 km). Most patients (76%) were insured by Clalit Health Services; 68% had public supplementary insurance.

The patients were diagnosed with lung (33%), breast (29%), gastric (9%) and pancreatic cancer (8%), with 39% defined as orphan diseases. The majority of the off-label requests were for patients with metastatic diseases (69%), who had received at least one prior treatment that had failed (53%); 25% had a molecular marker or mutation correlated with a biological plausibility for response. Off-label request were for chemotherapies (20%), targeted therapies (39%) and immunotherapies (41%). Only 48% of the requests had sufficient supporting evidence. The full description of the requests, patient diseases and socio-demographic characteristics are presented in Tables 1, 2.

**TABLE 1:** Off-label request patient characteristics and univariate logistic regression analysis for treatment initiation (*n* = 708).

Characteristic	Total *n* (%)	Treatment initiated *n* = 583 *n* (%)	Treatment not initiated *n* = 125 *n* (%)	Odds ratio	95% CI for odds ratio lower upper	*p*-value
Age (years) Median, Mean	64, 62 (19–95)	64, 62 (19–95)	65, 64 (27–89)	1.012	0.997–1.028	0.114
(range)						
Gender Male	297 (42)	243 (42)	54 (43)	1.064	0.720–1.572	0.755
Female	411 (58)	340 (58)	71 (57)			
Children	532 (75)	433 (74)	99 (79)	1.319	0.824–2.111	0.248
Health insurer						
Clalit	539 (76)	439 (75)	100 (80)	0.762	0.473–1.228	0.264
Maccabi	99 (14)	85 (14.4)	14 (11)	1.353	0.742–2.470	0.324
Meuhedet	39 (5.5)	30 (5)	9 (7)	0.699	0.323–1.512	0.363
Leumit	27 (4)	25 (5)	2 (2)	2.755	0.644–11.787	0.172
Tourist/Not insured	4 (0.5)	4 (0.5)	0 (0)	NA	NA	NA
Supplementary public insurance						
None	228 (32)	195 (33)	33 (26)	1.401	0.908–2.161	0.127
Basic	226 (32)	183 (32)	43 (34)	0.872	0.508–1.312	0.513
Premium	254 (36)	205 (35)	49 (40)	0.841	0.565–1.251	0.393
SES Median Decile	7	7	7	1.078	0.961–1.210	0.198
Decile 1–3	59 (8)	50 (9)	9 (7)			
Decile 8–10	264 (37)	217 (37)	47 (38)			
Distanceto RMC (km) Median, Mean (range)	18.5, 30 (3–344)	18.5, 30 (3–344)	15, 29 (3–344)	0.999	0.994–1.004	0.779


SES, Socioeconomic status; RMC, Rabin Medical Center; km, kilometers.

Gender is for Males compared to Females.

**TABLE 2:** Off-label request characteristics and univariate logistic regression analysis for treatment initiation (*n* = 708).

Characteristic	Total *n* (%)	Treatment initiated *n* = 583 *n* (%)	Treatment not initiated *n* = 125 *n* (%)	Odds ratio	95% CI for odds ratio lower upper	*p*-value
Drug type						
Chemotherapy	144 (20)	122 (21)	22 (18)	1.239	0.750–2.046	0.402
Targeted therapy	272 (39)	208 (36)	64 (51)	0.529	0.358–0.780	0.001
Immunotherapy	292 (41)	253 (43)	39 (31)	1.691	1.119–2.553	0.013
Metastatic disease	491 (69)	417 (72)	74 (59)	1.731	1.161–2.581	0.007
Orphan disease	276 (39)	227 (39)	49 (39)	0.989	0.666–1.469	0.956
Marker or targetable mutation	179 (25)	157 (27)	22 (18)	1.725	1.051–2.832	0.031
Treatment line						
≥2	377 (53)	318 (55)	59 (47)	1.342	0.911–1.977	0.136
≥3	109 (15)	90 (15)	19 (15)	1.018	0.595–1.743	0.947
Supporting evidence						
Sufficient	338 (48)	226 (45)	72 (58)	1.619	1.096–2.392	0.016
Limited	322 (45)	278 (48)	44 (35)	0.596	0.399–0.899	0.012
Inadequate	48 (7)	39 (7)	9 (7)	1.082	0.510–2.296	0.837
Financing						
Expanded access	210 (30)	187 (32)	23 (18)	2.094	1.290–3.400	0.003
Charity	17 (2)	16 (3)	1 (1)	3.499	0.440–26.632	0.226
Out-of-pocket	84 (12)	71 (12)	13 (10)	1.195	0.639–2.233	0.557
Private insurance	178 (25)	152 (26)	21 (21)	1.343	0.839–2.148	0.219
No planned source	219 (31)	157 (27)	62 (50)	0.374	0.252–0.556	<0.0005
Cost per month (ILS), Mean	54,703	56,274	47,313	1	1–1	0.125

ILS, New Israeli Shekels.

### Off-Label Treatments Cost and Financing Sources

The mean monthly cost of the planned treatment was ILS54,703 (SD = ILS61,487, median = ILS39,928) for all requests and ILS64,436 (SD = ILS58,066, median = ILS49,157) for metastatic diseases. The main planned sources for financing were private health insurance (25%) and expanded access pharma company plans (30%). However, a large proportion (31%) of the patients did not specify a source of reimbursement at the time of the off-label request.

**FIGURE 1:**
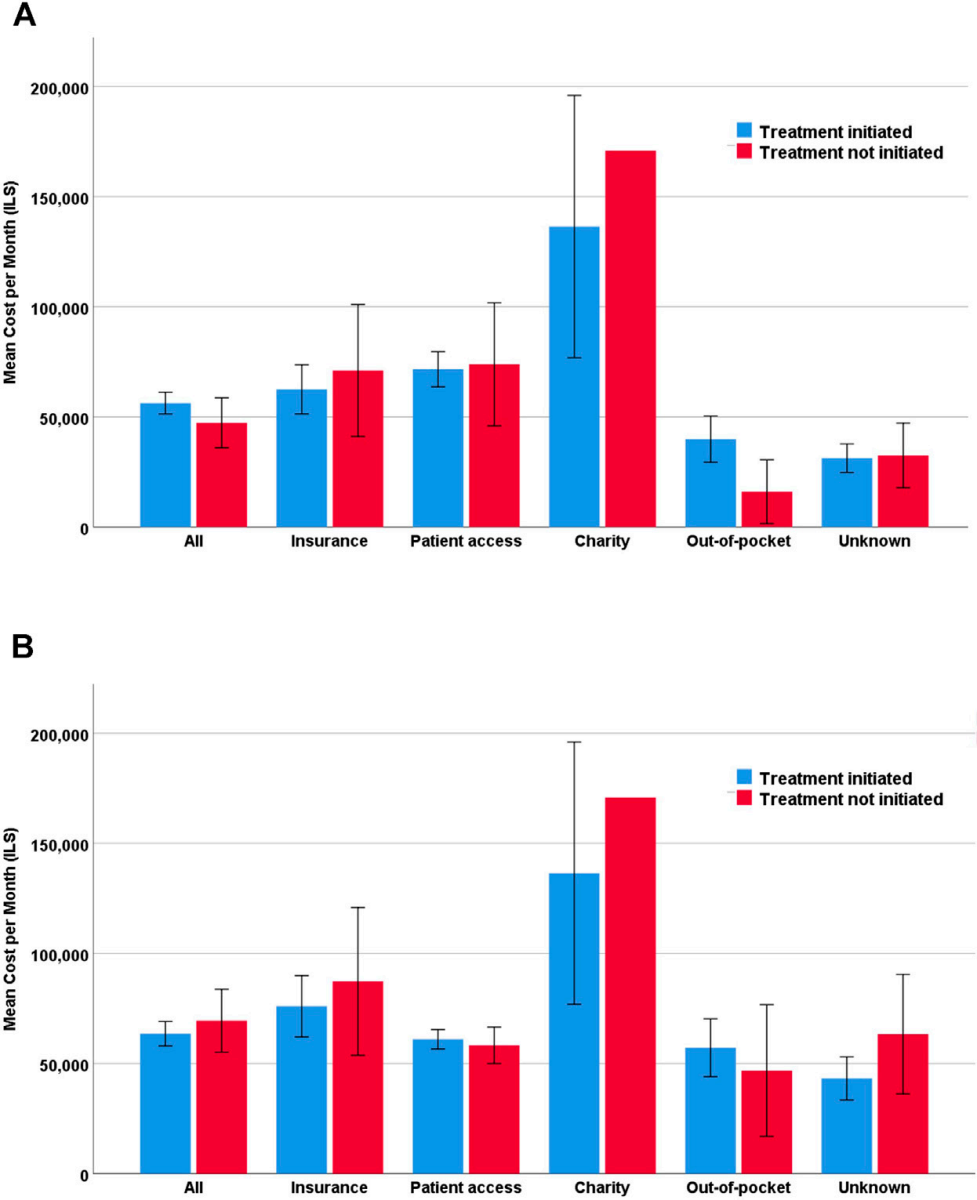
Mean monthly cost by financing source. **(A)** All off-label requests (*n* = 708) **(B)** Metastatic disease off-label requests (*n* = 491).

Of the 708 approved prescription requests, only 583 (82%) were initiated. The mean monthly costs were higher for treatments that were initiated compared to those that were not (ILS56,274 vs. ILS47,313); however, this trend was not statistically significant (Figure 1A). In the metastatic setting, the opposite trend was observed, with slightly, albeit not significantly, higher mean costs of treatments that were not initiated (ILS63,558 vs. ILS69,451) (Figure 1B). This trend persisted when further exploring metastatic setting requests by planned financing source. Treatments that were not initiated had higher mean monthly costs for private health insurance (ILS76,002 vs. ILS87,287) and when the financing source was unknown at the time of request submission (ILS43,222 vs. ILS63,290). Nevertheless, none of the trends were statistically significant (Figure 1B).

### Factors Associated With Off-Label Treatment Initiation

To identify the patient and request characteristics that influenced the likelihood that an approved off-label request would eventually be initiated, a univariate logistic regression analysis was conducted (Tables 1, 2). None of the patient characteristics were found to be predictive of treatment initiation. The disease and treatment characteristics that were significantly predictive of treatment initiation were metastatic disease (OR = 1.731; 95% CI, 1.161 to 2.581; *p* = 0.007) and the existence of a molecular marker or a targetable mutation which was related to a response to the treatment (OR = 1.725; 95% CI, 1.051 to 2.832; *p* = 0.031). Immunotherapy was predictive of treatment initiation (OR = 1.691; 95% CI, 1.119 to 2.553; *p* = 0.013), whereas targeted therapy was less likely to be initiated (OR = 0.529; 95% CI, 0.358 to 0.780; *p* = 0.001). A treatment was more likely to be initiated if it was planned to be sponsored by the pharma company through expanded access plans (OR = 2.094; 95% CI, 1.290 to 3.400; *p* = 0.003). By contrast, treatment initiation was less likely if no financing source was in place (OR = 0.374; 95% CI, 0.252 to 0.556; *p* < 0.0005).

A multivariate logistic regression was performed to ascertain the independent effects of age, gender, drug type, metastatic disease, marker or targetable mutation, supporting evidence level, and planned financing source on the likelihood that an approved off-label request would eventually be initiated. The model explained 12% (Nagelkerke *R* ^*2*^) of the variance in treatment initiation and correctly classified 82.1% of the cases. Of the potential predictor variables, only two were statistically significant: targeted therapy (OR = 0.407; *p* = 0.005) and unknown financing source (OR = 0.400; *p* < 0.0005) (Table 3). If the treatment was a targeted therapy or if no financing plan was in place at the time of treatment request, there was a 2.5-fold higher likelihood of not receiving treatment.

**TABLE 3:** Multivariate logistic regression predicting the likelihood of treatment initiation based on age, gender, drug type, metastatic disease, marker or targetable mutation, supporting evidence and planned financing source (*n* = 708).

	B	SE	Wald	df	*p*-value	Odds ratio	95% CI for odds ratio	
							Lower	Upper
Age	0.013	0.009	2.285	1	0.131	1.013	0.966	1.031
Gender
Male	0.458	0.252	3.292	1	0.700	1.580	0.964	2.591
Drug type								
Targeted therapy	−0.898	0.320	7.882	1	0.005	0.407	0.218	0.762
Immunotherapy	−0.221	0.378	0.340	1	0.560	0.802	0.382	1.683
Metastatic disease	0.092	0.322	0.082	1	0.775	0.912	0.485	1.715
Marker	0.275	0.277	0.991	1	0.320	1.317	0.766	2.265
Supporting evidence								
Sufficient	−0.086	0.458	0.035	1	0.852	0.918	0.374	2.252
Limited	0.659	0.444	2.203	1	0.138	1.933	0.810	4.617
Planned Financing Source								
Expanded access	0.511	0.304	2.829	1	0.093	1.668	0.919	3.026
No source	−0.916	0.262	12.252	1	<0.0005	0.400	0.240	0.668

Gender is for Males compared to Females.

## Discussion

This is the first study to evaluate the range of financing sources of oncology off-label drug usage within the context of a universal healthcare system. We found that the average monthly cost of off-label treatment was ILS54,703 and ILS64,436 in the metastatic setting. These costs were 4-5-fold higher than the net average (ILS15,751) household monthly income in Israel in 2016 (Central Bureau of Statistics, 2018).

The monthly cost was not found to predict treatment initiation but the results strongly suggest that cost plays a role in the approval process of commercial insurances, since treatments that were not initiated through this financing route were more expensive, although this result was not significant. Furthermore, treatments that did not have a planned financing source upfront and were not initiated eventually tended to be more expensive, implying that cost plays an important role in treatment initiation considerations.

A significant determinant that was found to predict whether a prescription was filled or not was external funding. If the treatment had no planned financing source at the time of approval, there was a 60% lower likelihood that the prescription would be filled. On the other hand, if the drug was provided through an expanded access plan, the odds that the prescription would be filled rose by 67%.

Several studies have estimated the prevalence of off-label prescriptions in oncology and examined patient, disease, and clinician predictors of off-label use and outcomes. These studies, however, have only analyzed off-label usage through drug dispensing records (Joerger et al., 2014; Hamel et al., 2015; Herrero Fernandez et al., 2019) or insurance claims (Conti et al., 2013; Eaton et al., 2016). Other studies have focused on supporting evidence of off-label use to identify hurdles to drug development for rare diseases (Bun et al., 2020). By contrast, this study focused on the costs and financing of treatments and examined requests for committee approval which also included information about the planned financing source. By comparing each off-label approval to the drug dispensing data, we were able to identify approved treatments that were not initiated. This enabled us to examine how funding influences the decision to initiate or forgo off-label treatment.

In Israel, there are various solutions for prescribing drugs outside their registered indications and for reimbursement within universal healthcare systems. Since 2008, supplementary health insurance plans in Israel, which are offered by the four public health insurers, are not allowed to cover “life-saving” or “life-extending” treatments but several alternatives are still available. For example, if the off-label treatment is in the process of registration, many pharmaceutical companies will fund an expanded access plan (Fountzilas et al., 2018). Moreover, despite universal healthcare with wide coverage, 35% of Israeli adults also have private commercial health insurance (Central Bureau of Statistics, 2019). These insurance plans may cover off-label treatments if registered in another country or accepted in clinical guidelines. Some patients and families are able to pay for off-label treatments out-of-pocket, but this creates great financial hardship, since costs in oncology are high and treatment duration can be long. Charities and aid organizations offer assistance for drug supply and financial support. Nevertheless, in many instances, no financing is available and patients forgo treatment.

A cross-sectional survey conducted in 2011 examined public experiences with financing therapies outside the National List of Health Services in Israel (Sperling, 2014). The requests for reimbursement from commercial insurance (23.3 vs. 25% in this study) and aid organizations (5.2 vs. 2%) were similar. However, in the current study only 12% of the requests were financed out-of-pocket, whereas the 2011 survey reported that 56.9% of all patients relied on private purchase. It is possible that during the time between the two studies, pharmaceutical companies expanded their early access plans and a higher proportion of the population acquired private commercial health insurance covering the costs of these drugs. In this study, 31% of the requests had no planned financing source. These patients eventually pay for their therapy privately (out-of-pocket), turn to charity organizations, or forgo treatment. In fact, patients in the current study were found to be four times more likely to forgo treatment than previously reported (17.7 vs. 4.3%). This study focused solely on oncology, where drugs are more expensive, out-of-pocket payments are less feasible, and forgoing treatment is more common. Furthermore, 69% of the patients were metastatic, and many of them turned to off-label use after exhausting all the available options. Forgoing active cancer treatment might be more common in the case of a terminal diagnosis.

This study has several limitations. First, it only dealt with injectable agents administered at the outpatient clinic in the oncology center. According to Israeli regulations, oral off-label therapies are handled at the regional Ministry of Health level and approved by the district pharmacist. Prescriptions are filled by private pharmacies that specialize in off-label treatments, including importing unlicensed drugs. Thus, the findings here may not be generalizable to other types of off-label therapies. Further research is needed to determine whether different costs and payment sources, disease or sociodemographic characteristics affect prescription filling in these settings.

Second, the analysis was restricted to anticipated or intended financing sources specified by the patient when the off-label request for approval was submitted. This explains the high percentage of patients who did not know how the treatment would be financed. Actual financing may have differed from expected financing due to treatment costs or duration of treatment. Moreover, treatment can be funded from more than one source. Capturing the patient’s intention and the actual prescription filling separately could shed light on this issue.

Finally, in Israel, which has a universal healthcare system, there is a mechanism of public reimbursement of off-label treatments through special exception committees within each of the four public health insurance plans. If an off-label therapy is determined to be effective for an individual patient, payment for ongoing treatment may be authorized regardless of how it was initially financed. To prove effectiveness and obtain reimbursement, the patient must have an initial financing source for the treatment. If this initial funding is unfeasible, many patients decide not to initiate a treatment that could have been effective. Unlike other studies, both delivered and undelivered off-label treatments and associated costs were identified here. However, data confirming that the expected funding source did in fact cover the cost of treatment were not analyzed. Further research is needed on complete financing and payment information over the entire course of the off-label treatment to draw conclusions.

Although novel mechanisms are authorized by regulatory agencies to facilitate scientific and clinical innovation in the development of new personalized therapies (Mullins et al., 2010), oncologists have even more rapid technology adoption patterns. Physicians want to give hope to their patients, and patients and families want to do everything in their fight against cancer. These unmet needs increase the demand for new treatments even if there is limited value. Once an off-label treatment is prescribed, patients and their family engage in a race to obtain funding for the therapy. Little is known about the financial burden patients and families undergo in this fight to get approval and secure funding for off-label treatments. This study described the costs of off-label drug use in oncology and sources of financing. The insights from this study with respect to identifying the hurdles to access off-label treatments may be utilized in further studies focusing on the feasibility and workability of financing solutions.

## Data Availability

Data are not publicly available according to the Rabin Medical Center strict institutional policy with regards to public availability of unidentified patient data. However, data that are minimally required to replicate the outcomes of this study will be made available upon request. Further inquiries can be directed to the corresponding author NG, noagordon@gmail.com.

## References

[B1] Abernethy A. P. Raman G. Balk E. M. Hammond J. M. Orlando L. A. Wheeler J. L. (2009). Systematic Review: Reliability of Compendia Methods for Off-Label Oncology Indications. Ann. Intern. Med. 150, 336–343. 10.7326/0003-4819-150-5-200903030-00107 19221366

[B2] ASCO (2006). Reimbursement for Cancer Treatment: Coverage of Off-Label Drug Indications. J. Clin. Oncol. 24 (19), 3206–3208. 10.1200/JCO.2006.06.8940 16717290

[B3] Bach P. B. (2009). Limits on Medicare's Ability to Control Rising Spending on Cancer Drugs. N. Engl. J. Med. 360 (6), 626–633. 10.1056/nejmhpr0807774 19176475

[B4] Bennette C. S. Richards C. Sullivan S. D. Ramsey S. D. (2016). Steady Increase in Prices for Oral Anticancer Drugs after Market Launch Suggests a Lack of Competitive Pressure. Health Aff. (Millwood) 35 (5), 805–812. 10.1377/hlthaff.2015.1145 27140986

[B5] Bun S. Yonemori K. Sunadoi H. Nishigaki R. Noguchi E. Okusaka T. (2020). Safety and Evidence of Off-Label Use of Approved Drugs at the National Cancer Center Hospital in Japan. JCO Oncol. Pract. 8, OP2000131. 10.1200/OP.20.00131 32956004

[B6] Central Bureau of Statistics (2018). Household Income and Expenditure Data from the 2016 Household Expenditure Survey General. Available at: https://www.cbs.gov.il/he/publications/DocLib/2019/1719/e_print.pdf .

[B7] Central Bureau of Statistics (2019). Selected Data on Health Insurances and Health Information from the 2017 Social Survey. Available at: https://www.cbs.gov.il/he/mediarelease/DocLib/2019/035/19_19_035b.pdf .

[B8] Cherny N. I. Dafni U. Bogaerts J. Latino N. J. Pentheroudakis G. Douillard J. Y. (2017). ESMO-magnitude of Clinical Benefit Scale Version 1.1. Ann. Oncol. 28 (10), 2340–2366. 10.1093/annonc/mdx310 28945867

[B9] Conti R. M. Bernstein A. C. Villaflor V. M. Schilsky R. L. Rosenthal M. B. Bach P. B. (2013). Prevalence of Off-Label Use and Spending in 2010 Among Patent-Protected Chemotherapies in a Population-Based Cohort of Medical Oncologists. J. Clin. Oncol. 31 (9), 1134–1139. 10.1200/JCO.2012.42.7252 23423747 PMC3595423

[B10] Eaton A. A. Sima C. S. Panageas K. S. (2016). Prevalence and Safety of Off-Label Use of Chemotherapeutic Agents in Older Patients with Breast Cancer: Estimates from SEER-Medicare Data. J. Natl. Compr. Canc. Netw. 14 (1), 57–65. 10.6004/jnccn.2016.0007 26733555 PMC4827612

[B11] Elkin E. B. Bach P. B. (2010). Cancer's Next Frontier: Addressing High and Increasing Costs. JAMA 303 (11), 1086–1087. 10.1001/jama.2010.283 20233828 PMC3647336

[B12] Fountzilas E. Said R. Tsimberidou A. M. (2018). Expanded Access to Investigational Drugs: Balancing Patient Safety with Potential Therapeutic Benefits. Expert Opin. Investig. Drugs 27 (2), 155–162. 10.1080/13543784.2018.1430137 PMC6291242 29353505

[B13] Garrison L. P. Kamal-bahl S. Towse A. (2017). Toward a Broader Concept of Value: Identifying and Defining Elements for an Expanded Cost-Effectiveness Analysis. Value Health 20 (2), 213–216. 10.1016/j.jval.2016.12.005 28237197

[B14] Garrison L. P. Pauly M. V. Willke R. J. Neumann P. J. (2018). An Overview of Value, Perspective, and Decision Context-A Health Economics Approach: An ISPOR Special Task Force Report [2]. Value Health 21 (2), 124–130. 10.1016/j.jval.2017.12.006 29477389

[B15] Goldstein D. A. Stemmer S. M. Gordon N. (2016). The Cost and Value of Cancer Drugs - Are New Innovations Outpacing Our Ability to Pay. Isr. J. Health Pol. Res. 5 (40), 40. 10.1186/s13584-016-0096-110.1186/s13584-016-0097-0 PMC5032240 27688873

[B16] Gordon N. Stemmer S. M. Greenberg D. Goldstein D. A. (2017). Trajectories of Injectable Cancer Drug Costs after Launch in the United States. J. Clin. Oncol. 36, 319–325. 10.1200/JCO.2016.72.2124 29016226

[B17] Gozzo L. Longo L. Vitale D. C. Drago F. (2020). The Regulatory Challenges for Drug Repurposing during the Covid-19 Pandemic: The Italian Experience. Front. Pharmacol. 11, 588132. 10.3389/fphar.2020.588132 33101042 PMC7546760

[B18] Green A. K. Wood W. A. Basch E. M. (2016). Time to Reassess the Cancer Compendia for Off-Label Drug Coverage in Oncology. JAMA 316 (15), 1541–1542. 10.1001/jama.2016.12770 27561002

[B19] Greenberg D. Siebzehner M. I. Pliskin J. S. (2009). The Process of Updating the National List of Health Services in Israel: Is it Legitimate? Is it Fair. Int. J. Technol. Assess. Health Care 25 (3), 255–261. 10.1017/S026646230999016X 19619343

[B20] Hamel S. McNair D. S. Birkett N. J. Mattison D. R. Krantis A. Krewski D. (2015). Off-label Use of Cancer Therapies in Women Diagnosed with Breast Cancer in the United States. SpringerPlus 4 (1), 209. 10.1186/s40064-015-0981-z 25977897 PMC4422830

[B21] Hammerman A. Lishitz Y. Phliskin Y. Greenberg D. (2011). Public Reimbursement of Off-Label Drug Use (Off-label Use). Harefua 150 (2), 163–167. 22164947

[B22] Herrero Fernandez M. Molina Villaverde R. Arroyo Yustos M. Navarro Expósito F. Lopez Gonzalez J. L. Luque Infantes M. R. (2019). The Off-Label Use of Antineoplastics in Oncology Is Limited but Has Notable Scientific Support in a university Hospital Setting. Front. Pharmacol. 10 (OCT), 1210. 10.3389/fphar.2019.01210 31708769 PMC6820060

[B23] Joerger M. Schaer-Thuer C. Koeberle D. Matter-Walstra K. Gibbons-Marsico J. Diem S. (2014). Off-label Use of Anticancer Drugs in Eastern Switzerland: A Population-Based Prospective Cohort Study. Eur. J. Clin. Pharmacol. 70 (6), 719–725. 10.1007/s00228-014-1662-5 24609468

[B24] Kalis J. A. Pence S. J. Mancini R. S. Zuckerman D. S. Ineck J. R. (2015). Prevalence of Off-Label Use of Oral Oncolytics at a Community Cancer Center. J. Oncol. Pract. 11 (2), e139–43. 10.1200/jop.2014.001354 25604593

[B25] Mateo J. Chakravarty D. Dienstmann R. Jezdic S. Gonzalez-Perez A. Lopez-Bigas N. (2018). A Framework to Rank Genomic Alterations as Targets for Cancer Precision Medicine: The ESMO Scale for Clinical Actionability of Molecular Targets (ESCAT). Ann. Oncol. 29 (9), 1895–1902. 10.1093/annonc/mdy263 30137196 PMC6158764

[B26] Ministry of Health (2016). Prescription Drugs Price List [Hebrew]. Jerusalem: Ministry of Health. Available at: https://www.health.gov.il/Subjects/Finance/DrugPrice/Pages/default.aspx (Accessed August 26, 2020).

[B27] Mullins C. D. Montgomery R. Tunis S. (2010). Uncertainty in Assessing Value of Oncology Treatments. Oncologist 15 Suppl 1 (S1), 58–64. 10.1634/theoncologist.2010-s1-58 20237219

[B28] Natz A. Campion M.-G. (2012). Pricing and Reimbursement of Innovative Pharmaceuticals in France and the New Healthcare Reform. Fe 13 (4), 49–60. 10.7175/fe.v13i2.270

[B29] Rawson N. Chhabra A. (2018). Public Reimbursement of Prescription Drugs Used for Off-Label Indications in Ontario. J. Popul. Ther. Clin. Pharmacol. 25 (2), e23–e30. 10.22374/1710-6222.25.2.3 30725540

[B30] Ribalov R. Morginstin T. Greenberg D. (2016). Are We Indeed Fast Adopters as We Claim to Be? an Analysis of the Time Interval from Regulatory Approval to Coverage and Reimbursement in the National List of Health Services in Israel. Value in Health 19, A447. 10.1016/j.jval.2016.09.582

[B31] Saiyed M. M. Ong P. S. Chew L. (2017). Off-label Drug Use in Oncology: a Systematic Review of Literature. J. Clin. Pharm. Ther. 42, 251–258. 10.1111/jcpt.12507 28164359

[B32] Shani S. Siebzehner M. I. Luxenburg O. Shemer J. (2000). Setting Priorities for the Adoption of Health Technologies on a National Level – the Israeli Experience. Health Policy 54, 169–185. 10.1016/s0168-8510(00)00109-3 11154787

[B33] Sperling D. (2014). Needs, Expectations and Public Knowledge Concerning Services outside the Medical Basket: A Lesson from Israel. Health Policy 117 (2), 247–256. 10.1016/j.healthpol.2014.03.004 24746695

[B34] Weda M. Hoebert J. Vervloet M. Moltó Puigmarti C. Damen N. Marchange S. (2017). Study on Off-Label Use of Medicinal Products in the European Union. Brusseles: European Union. Available at: https://ec.europa.eu/health/sites/default/files/files/documents/2017_02_28_final_study_report_on_off-label_use_.pdf .

[B35] Wittich C. M. Burkle C. M. Lanier W. L. (2012). Ten Common Questions (And Their Answers) about Off-Label Drug Use. Mayo Clin. Proc. 87 (10), 982–990. 10.1016/j.mayocp.2012.04.017 22877654 PMC3538391

